# The association between parent-child relationship and problematic internet use among English- and Chinese-language studies: A meta-analysis

**DOI:** 10.3389/fpsyg.2022.885819

**Published:** 2022-08-29

**Authors:** Yalin Zhu, Linyuan Deng, Kun Wan

**Affiliations:** ^1^Faculty of Education, Beijing Normal University, Beijing, China; ^2^Nanhai Middle School of Nanshan Experimental Educational Group, Shenzhen, China

**Keywords:** problematic internet use, parent-child relationship, meta-analysis, Internet addiction, parent-child attachment, English-and Chinese-language studies

## Abstract

As past studies of the association between parent-child relationship and problematic internet use show mixed results and are influenced by many factors, this meta-analysis of 75 primary Chinese and English language studies from 1990 to 2021 with 110,601 participants (aged 6−25 years) explored (a) the overall association between parent-child relationship and problematic internet use, and (b) whether the association is affected by their types, country, measures, objects of the parent-child relationship, gender, age, year and publication types. We used funnel plots, *Classic fail-safe N* and *Egger's test* to test for publication bias and for moderation with the homogeneity tests. The results showed a negative association between quality of parent-child relationship and problematic internet use (*r* = −0.18, 95% CI = [−0.20, −0.15]). The moderation analysis found that compared with internet addiction tendency, the association between social media addiction and parent-child relationship was stronger. Moreover, the association between the parent-child relationship and problematic internet use of emerging adults (18–25 years old) was stronger than that of adolescents (12−18 years old). Furthermore, the negative association between parent-child relationship and problematic internet use was weaker (a) in Italy than those in Turkey and China, (b) when using CPS (Closeness to Parents Scale), IPPA (Inventory of Parent and Peer Attachment), or PARQ (Parent-Child Relationship Questionnaire) measuring parent-child relationship than using PCCS (Parent-Child Communication Scale), (c) when using IAT measuring problematic internet use rather than using IGDS or APIUS. Hence, these results indicate a negative association between parent-child relationships and problematic internet use, and the association is moderated by types of problematic internet use, age, country, scales of both parent-child relationship and problematic internet use.

## Introduction

With the rapid development of information technology and lifestyle changes, the number of Internet users is increasing (Fumero et al., 2018), and the communication among people has gradually changed from offline to online (Billieux, 2012). As of January 2021, the global Internet penetration rate reached 59.5%, and the number of mobile phone users accounted for 66.6% of the global population. Due to the outbreak of COVID-19, the global social media penetration rate increased by more than 13% in the past, accounting for more than 53% of the global population (Kemp, 2021). According to the 47th statistical report on Internet development in China, the number of netizens in China had reached nearly 1 billion by December 2020, with a penetration rate of 70.4%, and 99.7% of them use mobile phones to access the Internet. China is becoming the largest “digital country” in the world, and the majority of Chinese netizens are gradually spreading to minors and the elderly (Center CINI, 2021). Internet-related problems (e.g., addiction, abuse) have become a global public health issue (Shao et al., 2018), and the global Internet addiction rate is on the rise (Weinstein and Lejoyeux, 2010; Shao et al., 2018).

Researchers used different terms to define *problematic Internet use* (Pontes and Griffiths, 2015). Young first used the term Internet Addiction to define problematic Internet use (Young, 1998), emphasizing uncontrollable use of the Internet and the adverse effects on an individual's physical and mental health. In this study, problematic Internet use will refer to problems associated with Internet addiction (Say and Batigun, 2016). Problematic Internet use is related to a range of psychological and behavioral problems at the individual level, such as attention deficit disorder (Chan and Rabinowitz, 2006), depression and anxiety (King et al., 2013), obsessive-compulsive personality disorder (Ballarotto et al., 2018), aggressive tendencies (Kim et al., 2008), having an impact on an individual's sleep, diet, and physical activity, lead to loneliness (Baturay and Toker, 2019). In addition, problematic Internet use may also impact their social adaptation (Pies, 2009), such as avoiding life and learning responsibilities, avoiding offline social activities, and affecting adolescents' academic performance and interpersonal relationships (Mahmood, 2010).

*Parent-child relationship* is an important interpersonal relationship in one's life (Zhen et al., 2019), which usually refers to the relationship between parents (primary caregivers, which can be non-biological parents) and children (Schneider et al., 2017). It is the first established relationship and multi-dimensional structure (Abar et al., 2014) with good or bad quality. Surveys on interpersonal troubles between Chinese (Quan, 2008) and American students (Abar et al., 2014) found that the most problematic interpersonal relationship is the parent-child relationship. Bad parent-child relationships will lead to internalization and externalization problems in children and adolescents (Achenbach and Dumenci, 2001). Internalization problems are mainly emotional disorders, such as anxiety, depression (Zhou et al., 2021), loneliness and other emotional problems (Lee and Hankin, 2009). Externalization problems mainly include behavioral problems, such as aggressive behavior (Wang, 2017), social problems, moral problems, behavioral disorders, etc. (Cai and Zhou, 2006).

In general, both parent-child relationship and problematic Internet use have a significant impact on individuals. There is a strong link between the parent-child relationship and problematic Internet use. Social support theory (Cutrona et al., 1994) believes that a lack of social relations (e.g., parent-child relationship) may be more likely to produce problematic Internet use (Ballarotto et al., 2018; Ceyhan et al., 2018; Fumero et al., 2018). Social connection theory suggests (Koeppel and Chism, 2018) that the parent-child relationship as a solid social support force significantly impacts problematic Internet use (Lyu, 2017; Zhen et al., 2019). This study will explore (a) the overall association between problematic Internet use and parent-child relationship and (b) whether the association is influenced by types of problematic Internet use, types of parent-child relationship, country, scales, objects of parent-child relationship, gender, age, year of literature, types of literature publication.

## Problematic internet use and parent-child relationship

We will discuss the association between problematic Internet use and the quality of parent-child relationship in terms of main and moderating effect.

### Analysis of main effect

First, we discuss why we will explore the overall relationship between problematic Internet use and the quality of parent-child relationship. Many studies have shown that family is the crucial factor leading to problematic Internet use (Li et al., 2014; Schneider et al., 2017). The parent-child relationship has a greater impact on adolescents' game use (Schneider et al., 2017) and adolescents' mobile phone addiction than the teacher-student relationship (Zhen et al., 2019). The survey of children (Huang, 2018) and college students (Chen et al., 2016) also found that poor parent-child relationships would lead to Internet addiction. Good parent-child attachment can make adolescents feel accepted (Deng et al., 2013b). It is generally believed that a good parent-child relationship is a protective factor against Internet addiction (D'Arienzo et al., 2019), while parent-child conflict will aggravate Internet addiction (Lyu, 2017; Ballarotto et al., 2018; Huang et al., 2019; Bilgin et al., 2020). A large number of longitudinal studies have also shown that a poor parent-child relationship is an important cause of problematic Internet use. Szwedo et al. conducted a 7-year follow-up study on 138 adolescents and found that children with poor mother-child relationships prefer online communication and establish social relations through the Internet (Szwedo et al., 2011). Shek et al. (2019) conducted a 3-year follow-up study on 3,328 7th grade Hong Kong students. They found that the better the quality of parent-child relationship, the faster the decline of Internet addiction. Internet addicts communicate less with their families and are more likely to have family problems (Li et al., 2014). The results of a meta-analysis also showed that social media use can negatively affect offline family communication (Tadpatrikar et al., 2021). The social function of people with long-term Internet addiction will further deteriorate, leading to more internalized problems, such as suicide and depression (Padilla-Walker et al., 2020).

At present, no study has comprehensively compared the relationship between different types of problematic Internet use and different types of parent-child relationship. Therefore, the first purpose of the study is to explore the overall association between problematic Internet use and parent-child relationship.

### Moderation: Types of problematic internet use, types of parent-child relationship, country, measures, object of parent-child relationship, participant characteristics (gender, age) and literature types (year, types of literature publication)

Second, we will discuss the moderation of some critical variables. The association between problematic Internet use and parent-child relationship may be affected by types of problematic internet use, types of parent-child relationship, countries, scales, parental identity, participant characteristics (gender, age) and literature types (year, types of literature publication), see Table 1 for moderating variables.

**Table 1:** Types of moderating variables.

**Types of problematic internet use**	**Types of parent-child relationship**	**Age**	**Object of parent-child relationship**	**Measures of parent-child relationship**	**Measures of problematic internet use**	**Types of literature**
Excessive Internet use (EIU)	Parent-child attachment (PCA)	Children	Parents	Closeness to Parents Scale (CPS)	Adolescent Pathological Internet Use Scale (APIUS)	Journal article
General addiction (GA)	Parent-child communication (PCC)	Adolescents	Mother	Family Adaptation and Cohesion Evaluation Scales (FACES)	Chen Internet Addiction Scale (CIAS)	Thesis
Internet game addiction (IGA)	Parent-child conflict (PCF)	Emerging adult	Father	Inventory of Parent and Peer Attachment (IPPA)	Internet Addiction Test (IAT)	
Mobile phone addiction (MPA)	Parent-child affinity (PCI)			Parent-Adolescent Child Relationship Questionnaire (PACRQ)	Internet Gaming Disorder Scale (IGDS)	
Social media addiction (SMA)	Parent-child relationship (PCR)			Parent-Child Relationship Questionnaire (PARQ)	Mobile Phone Problem Use Scale (MPPUS)	
				Parent-Child Communication Scale (PCCS)	Others	
				Others		

#### Types of problematic internet use

The definition and terminology of problematic Internet use are inconsistent in different research (Fumero et al., 2018). The most typical term is Internet addiction (IA). Some studies have listed IA as behavioral addiction (Durkee et al., 2016) or impulse control disorder (Shapira et al., 2003). IA is mainly characterized by uncontrolled use of the Internet, impaired physical and psychological functions, individual behavior disturbance, and interference with normal life (Islam and Hossin, 2016; Atwood et al., 2017). Some researchers also call IA Pathological Internet use (PIU) or compulsive Internet use (CIU) (Atwood et al., 2017; Ballarotto et al., 2018; Zhao et al., 2018). Although PIU and IA are both problematic Internet use, there are differences in degree (Zhou et al., 2018). IA emphasizes the general adaptive impact of problematic Internet use, and PIU emphasizes the pathological results of Internet use, such as negative cognitive and behavioral patterns (Davis, 2001). Some researchers argue that the term IA may cause moral panic, and the term PIU suggests underlying neurobiological mechanisms (Yang et al., 2013). CIU emphasizes different Internet activities (Cacioppo et al., 2019). Furthermore, some researchers used excessive Internet use (EIU) to predict IA (Casal and Escario, 2019), but there are differences between EIU and IA (Ceyhan et al., 2018).

With the popularity of mobile phones, which have become indispensable to individuals, and further developed the mobile phone use related problems. The most typical is mobile phone dependence (MPD) (Kim and Chun, 2018), which is characterized by the interference of mobile phone use in daily life, habitual communication through mobile phone, tolerance to mobile phone use, and social interaction withdrawal (Kim et al., 2020), and has a certain tendency of addiction. As the symptoms evolve, mobile phone addiction (MPA) develops (Bae, 2015), that is, the uncontrollable excessive and forced use of mobile phones. In addition, among a series of problems caused by mobile phone use, problematic mobile phone use (PMPU) (Santana-Vega et al., 2019) is also included. Furthermore, almost everyone uses the Internet to socialize and work, developing social media addiction (SMA) (Bilgin et al., 2020). Facebook addiction (FA) is one of the specific types of SMA (Badenes-Ribera et al., 2019; Marino et al., 2019). In addition, more people relax through Internet games and further develop Internet Game Addiction (IGA), which is a typical problematic Internet use (Ceyhan et al., 2018). IGD is the only type of problematic Internet use to be included in the DSM-5.

These different terms further makes it difficult to unify the definition of problematic Internet use (Fumero et al., 2018). It is generally considered that it has the characteristics of frequent use, tolerance, withdrawal reaction and adverse consequences (Shao et al., 2018; Baturay and Toker, 2019), but there is no consensus on the characteristics of withdrawal and maladjustment (Fumero et al., 2018). From the perspective of the development process of problematic Internet use, if the EIU is not adjusted in time, it may intensify over time and eventually develop into IA (Caplan, 2003). The development of problematic Internet use includes the beginning, intermediate and final stages. As the stage goes on, the symptoms get worse. However, long-term Internet use is not necessarily an addictive behavior, and it is difficult to distinguish the relationship among them (Ceyhan et al., 2018). Previous studies have not strictly distinguished these problematic Internet use, which needs to be further explored. Researchers also did not strictly distinguish the definitions of these concepts. Therefore, the second purpose is to explore whether the association between problematic Internet use and parent-child relationship is moderated by the problematic Internet use types.

#### Types of parent-child relationship

Parental accepting-rejection theory emphasizes the role of parental acceptance and rejection in promoting and hindering adolescent mental health (Fard et al., 2016). However, there are few studies on the multiple aspects of the parent-child relationship at present. In general, there are two methods for evaluating the parent-child relationship (Li et al., 2014): The first is to evaluate the quality of the parent-child relationship directly by questionnaire, and the second is to predict the quality of the parent-child relationship through the influencing factors of parent-child relationship (such as parent-child communication, parent-child attachment, parent-child conflict, family harmony, etc.). However, the current family structure is shrinking, and most families live in the nuclear family (Cowan and Cowan, 2019). The parent-child subsystem is only a part of the whole family system (O'Gorman, 2012). The assessment of the quality of the overall family relationship and the quality of the husband-wife subsystem cannot directly represent the quality of the parent-child subsystem. Studies have also shown that variables directly related to the parent-child subsystem, such as parent-child communication (Lei et al., 2001), parent-child attachment (Yang et al., 2016), and parent-child conflict (Guo et al., 2018), can directly predict the quality of the parent-child relationship. Therefore, this study only uses the variables directly related to the parent-child subsystem (such as parent-child communication and parent-child attachment) to predict the quality of the parent-child relationship.

This study will combine multiple aspects of the parent-child relationship with exploring the parent-child relationship from direct and indirect perspectives, which is conducive to deepening the understanding of the parent-child subsystem. Therefore, the third purpose is to explore whether the association between problematic Internet use and parent-child relationship is moderated by parent-child relationship types.

#### Country

There may be differences in the detection rate of problematic Internet use in different cultural backgrounds. For college students, the results of a meta-analysis show that the detection rate of IA among Chinese college students (11%) is higher than that in other countries, and shows an upward trend (Shao et al., 2018). A cross-cultural study also shows that the IA and SMA rates of Chinese college students are higher than those of American and Singapore college students. However, the IGA rate of American college students is the highest (Tang et al., 2017). For adolescents, the prevalence of IA among adolescents in Europe and the United States ranges from 7.9 to 25.2%, and that in the Middle East and Africa ranges from 17.3 to 23.6%, while the prevalence of IA among adolescents in Asian countries varies greatly, ranging from 8.1 to 50.9% (Xin et al., 2018). A meta-analysis of the IA rate of Chinese adolescents shows that the IA rate of Chinese adolescents is 10% (Bian et al., 2016).

Different countries have different cultures. According to the cultural value model (Ho et al., 2008), the association between parent-child relationship and problematic Internet use may differ in different countries due to the influence of family patterns, cultural adaptation, economics and other factors (Cheung et al., 2014). First, different countries have different educational pressures. China's education is highly competitive, and parents want their children to be admitted to good schools. Teenagers have no place to vent their learning pressure, so they seek happiness and support on the Internet, aggravating their Internet addiction (Lim, 2012; Cheung et al., 2014; Zhen et al., 2019). Secondly, there are differences in family education patterns in different countries. Different family education will have different impacts on problematic Internet use. For example, family education in the United States pays more attention to exploring children's potential, realizing self-worth, cultivating independence consciousness and being more liberal and democratic (Huang and Huang, 2009). At the same time, European and American parents are more likely to use authoritative education, have specific standards of behavior and not overindulge. However, Chinese (eastern culture) family education focuses on intellectual education, pays too much attention to examination results, and ignores the overall development of children (Huang and Huang, 2009). In addition, Chinese parents tend to control their children's life and adopt an authoritarian parenting style while caring for them (Huang and Leung, 2009; Huntsinger and Jose, 2009; Yang et al., 2013).

The same type of family education has different effects in different countries. Different from western culture, Chinese culture is more compatible with authoritarian parenting. In China, authoritarian parents can help children achieve good results in school and work (Pong and Chen, 2010; Chan and Koo, 2011). However, other studies have found (Cheung et al., 2014) that children are more likely to have behavioral problems in a stressful family environment. A longitudinal survey of children and adolescents of different races shows that the harsher Canadian parents are, the more aggressive children are (Ho et al., 2008). Aggression is a significant predictor of problematic Internet use (Kim et al., 2018). Finally, different countries have differences in socioeconomic status (Ho et al., 2008). The higher the socioeconomic status, the less likely the child is to have problematic Internet use and the more positive the parenting style (Dong et al., 2019).

Different countries under the Eastern culture may also have subtle differences in culture. For example, The degree of parental control is higher in Japan than in China, while the degree of parental care is lower than that in China (Yang et al., 2013). Overall, parents greatly influence their children's internalization and externalization problems in eastern culture. There are more studies on the association between parent-child relationship and problematic Internet use (Kim et al., 2016). Moreover, as for parent-child communication, Chinese culture advocates non-confrontational communication, which is more implicit and indirect, tends to maintain the dignity of both sides in communication, and is easier to cooperate and compromise. This type of communication is manipulation and confusion, which negatively impacts problematic Internet use (Kim and Chong, 2005). However, according to the ethnic equivalence model (Eichelsheim et al., 2010), the relationship between parenting style and children's problem behaviors is similar in different countries.

It can be found that the association between parent-child relationship and problematic Internet use may be affected by different countries. Therefore, the fourth purposes to explore whether the association between problematic Internet use and parent-child relationship is moderated by the countries.

#### Scales

There are many scales for measuring problematic Internet use (Su et al., 2014), different scales have different diagnostic criteria (Su et al., 2014; Chi et al., 2016), and strict diagnostic criteria can reduce the detection rate of problematic Internet use. Different types of problematic Internet use can be divided into general and special addiction measurement scales (such as mobile phone addiction and Internet game addiction). At present, the general measurement scales of problematic Internet use mainly include Internet Addiction Test (IAT) (Young, 1998), Chen Internet Addiction Scale (CIAS) (Chen et al., 2003) and Adolescent Pathological Internet Use Scale (APIUS) (Lei and Yang, 2007). The primary measurement scale for mobile phone addiction is the Mobile Phone Problem Use Scale (MPPUS) (Bianchi and Phillips, 2005); The main measurement scale for online games is the Internet Gaming Disorder Scale (IGDS) (Pontes and Griffiths, 2015). In the general measurement scales of problematic Internet use, compulsive use was investigated more (79%), followed by negative outcomes (86%) and significantly more use of the Internet (71%), while escapism (21%), withdrawal symptoms (36%) and other aspects were less common (Lortie and Guitton, 2013). At present, there are still many self-compiled scales that lack effective verification approaches. Most researchers still use these generally recognized scales (Su and Lin, 2014), but problems still exist in the application.

According to the measurement method of parent-child relationship, it can be divided into direct measurement scales, such as Parent-Child Relationship Questionnaire (PARQ) (Stattin and Kerr, 2000), Parent-Adolescent Child Relationship Questionnaire (PACRQ) (Furman and Buhrmester, 1985) and Closeness to Parents Scale (CPS) (Buchanan et al., 1991); And indirect measurement scales, such as Parent-Child Communication Scale (PCCS) (Barnes and Olson, 1985), Family Adaptation and Cohesion Evaluation Scales (FACES) (Olson et al., 1979) and Inventory of Parent and Peer Attachment (IPPA) (Armsden and Greenberg, 1987). Each scale has a different emphasis (Chen, 2012). Hence, the fifth purpose is to explore whether the association between problematic Internet use and parent-child relationship is moderated by the scales of parent-child relationship and problematic Internet use.

#### Parental identity in the parent-child relationship

Few studies have distinguished the impact of father-child and mother-child relationships on problematic Internet use. Overall, both parent-child relationships are essential for individual health (D'Arienzo et al., 2019). A good relationship with either parent is a protective factor for children's problematic Internet use, but differences may exist.

Specifically, a poor father-child relationship was more likely to result in problematic Internet use than a poor mother-child relationship (Huang and Leung, 2009). Father's positive attitude (Zhou et al., 2018) toward Internet use and low paternal involvement in childhood (Rehbein and Baier, 2013) may exacerbate problematic Internet use. However, mothers' attitudes toward Internet use do not lead to problematic Internet use (Koh et al., 2009). Mothers are more likely to adopt psychological control to deal with children's problematic Internet use, while fathers are more likely to adopt behavioral control (Koh et al., 2009). Psychological control has a negative effect on problematic Internet use, while behavioral control has a positive effect on problematic Internet use (Shek et al., 2018; Cetinkaya, 2019).

The mother-child relationship in IGA addicts is better than the father-child relationship, but overall, the parent-child relationship is worse than that of ordinary people (Charlie et al., 2011). Other studies have found that father-child communication is not significantly correlated with IGA (Kim and Kim, 2015). A longitudinal study of early adolescents (Shek et al., 2019) shows that high paternal behavioral control can lead to increased problem behaviors. In addition, the combination of mothers' authoritarian parenting styles and fathers' neglectful parenting styles can increase the detection rate of problematic Internet use (Lukavska et al., 2020). It may be related to the division of power roles in the family. In the family, technology-related education is often dominated by the father, while emotion-related education is dominated by the mother (Zhou et al., 2018). In addition, father-child intimacy can improve children's self-control ability (Zhang et al., 2021), and the worse their self-control ability is, the higher the possibility of problematic Internet use (Atwood et al., 2017). Different parenting styles of fathers and mothers can lead to different influences on problematic Internet use.

Hence, the sixth purpose is to explore whether the association between problematic Internet use and parent-child relationship is moderated by the objects of parent-child relationship.

#### Participant characteristic

##### Gender

There may be gender differences in problematic Internet use. Boys generally have higher rates of problematic Internet use than girls (Fumero et al., 2018; Hsieh et al., 2018; Shao et al., 2018; Zhao et al., 2018). Regarding the motivation for Internet use, boys generally prefer to play online games, while girls prefer to use the Internet for social communication (Tang et al., 2017). Studies have found that parents are more tolerant of boys' problematic Internet use (Chou and Lee, 2017) and more inclined to adopt behavior control (Cetinkaya, 2019). In addition, parent-child intimacy has a greater impact on boys' problematic Internet use than girls (Schneider et al., 2017). Other studies have found that parents have different paths of influence on the problematic Internet use of boys and girls (Qin-Xue et al., 2013). For girls, the parent-child relationship has a greater impact on their problematic Internet use. Moreover, boys and girls also have different emphases on parent-child relationship (Awaluddin et al., 2019), and boys are more sensitive to parent-child conflict (Bush et al., 2013). Studies have also found that there is no gender difference in the association between parent-child relationship and Internet addiction (Yen et al., 2009). Different parenting styles of parents' responses to problematic Internet use in boys and girls and how boys and girls respond to them may have an impact on the strength of the link between parent-child relationship and problematic Internet use. Therefore, the seventh purpose is to explore whether the association between problematic Internet use and parent-child relationship is moderated by gender.

##### Age

Researches on the association between parent-child relationship and problematic Internet use mainly focused on children, adolescents, and college students. During childhood, their cognitive function develops continuously, and parents' cultivation and guidance have an important influence on their Internet use (Wu et al., 2014; Hsieh et al., 2020). During adolescence, the prefrontal cortex and limbic system are less mature and are more likely to engage in risk-taking and impulsive behavior (Ballarotto et al., 2018). The huge attraction of the Internet makes them easier to become addicted to it (Zhang, 2006). A Chinese literature review shows that problematic Internet use is common among teenagers, and the addiction rate is declining with age (Liu and He, 2019). However, a global survey shows that the IA rate among teenagers (4.6–4.7%) is lower than among college students (13–18.4%), and college students are more likely to become addicted to the Internet than teenagers (Young et al., 2011). The possible reason is that adolescents need more autonomy and independence to be psychologically separated from their parents (Ballarotto et al., 2018). At the same time, the authoritarian parenting style of Chinese parents has accelerated the emergence of problematic Internet use among teenagers (Cheung et al., 2014). College students are in their late adolescence (emerging adulthood), they need to redefine their personal, social and sexual identities (Ballarotto et al., 2018), and the quality of parent-child relationship can reflect how well adolescents transition to autonomy and adulthood (Ryan and Lynch, 1989).

In conclusion, the parent-child relationship changes with age, from childhood to adulthood, and the strength of the association with problematic Internet use may also change. Therefore, the eighth purpose is to explore whether the association between problematic Internet use and parent-child relationship is moderated by age.

#### Literature type

##### Year of literature publication

With the continuous development of the Internet, the incidence of problematic Internet use is also increasing. Both individual and social factors influence problematic Internet use. A meta-analysis found that personal factors (e.g., depression, anxiety and hostility, low self-esteem, emotional personality) had a greater impact on problematic Internet use than social factors (e.g., family factors, peer environment). Among social factors, parent-child relationship and peer relationship had a more significant impact on problematic Internet use (Fumero et al., 2018). Although parent-child relationship and problematic Internet use affect each other, as social life becomes more complicated, the association between the two may weaken. Therefore, the ninth purpose is to explore whether the association between problematic Internet use and parent-child relationship changes over time.

##### Types of literature publication

Journal articles and conference papers are usually more significant in results and more formal in style than the thesis. However, the thesis is valuable as long as it is based on writing norms. Publication bias may exist if only publications such as journal articles and conference papers are included (Viechtbauer, 2007). The literature types included in the meta-analysis should be as comprehensive as possible to ensure comprehensiveness. Therefore, the tenth purpose is to explore whether the association between problematic Internet use and parent-child relationship is moderated by types of literature publication.

## Materials and methods

### Literature search

This meta-analysis included Chinese- and English-language studies from January 1990 to March 2021. The first search began on October 14, 2020, and ended on October 16, 2020. The second search began on March 5, 2021, and ended on March 6, 2021.

First, the retrieval of the Chinese-language studies was carried out from the following three databases. The topics of “parent-child conflict (亲子冲突)”, “parent-child affinity (亲子亲和)”, “parent-child attachment (亲子依恋)”, “parent-child communication (亲子沟通)”, “parent-child relationship (亲子关系)”, “mobile phone addiction (手机成瘾)”, “internet game addiction (网络游戏成瘾)”, “internet addiction (网络成瘾)”, “mobile phone dependence (手机依赖)”, “pathological internet use (病理性网络使用)”, “excessive internet use (网络过度使用)”, “compulsive internet use (强迫性网络使用)”, “problematic social media use (问题性社交媒体使用)”, “social media addiction (社交媒体成瘾)” and “problematic mobile phone use (手机使用问题)” were searched in the following Chinese databases. The China National Knowledge Infrastructure (CNKI) retrieved 215 papers, and the China Science and Technology Journal Database retrieved 91 studies. Wanfang Data Knowledge Service Platform retrieved 144 studies, a total of 450 studies.

Second, the topics retrieved in the following seven English databases were “parent-child conflict”, “parent-child affinity”, “parent-child attachment”, “parent-child communication”, “parent-adolescent communication”, “parent-child relationship”, “parental Attachment”, “parent-adolescent relationship”, “parents” phubbing”, “mobile phone addiction”, “internet game addiction”, “internet addiction”, “mobile phone dependence”, “pathological internet use”, “excessive internet use”, “compulsive internet use”, “Facebook addiction”, “social media addiction”, “problematic social media use” and “problematic mobile phone use”. Web of Science (SCI) retrieved 298 literature articles, Proquest retrieved 44 articles, Science direct retrieved 94 articles, PsycNet (APA) retrieved 29 articles, SAGE retrieved 3567 articles, JSTOR retrieved 599 articles, and Scopus retrieved 49 articles, a total of 4680 literature articles.

### Inclusion and exclusion criteria for literature selection

**Figure 1:**
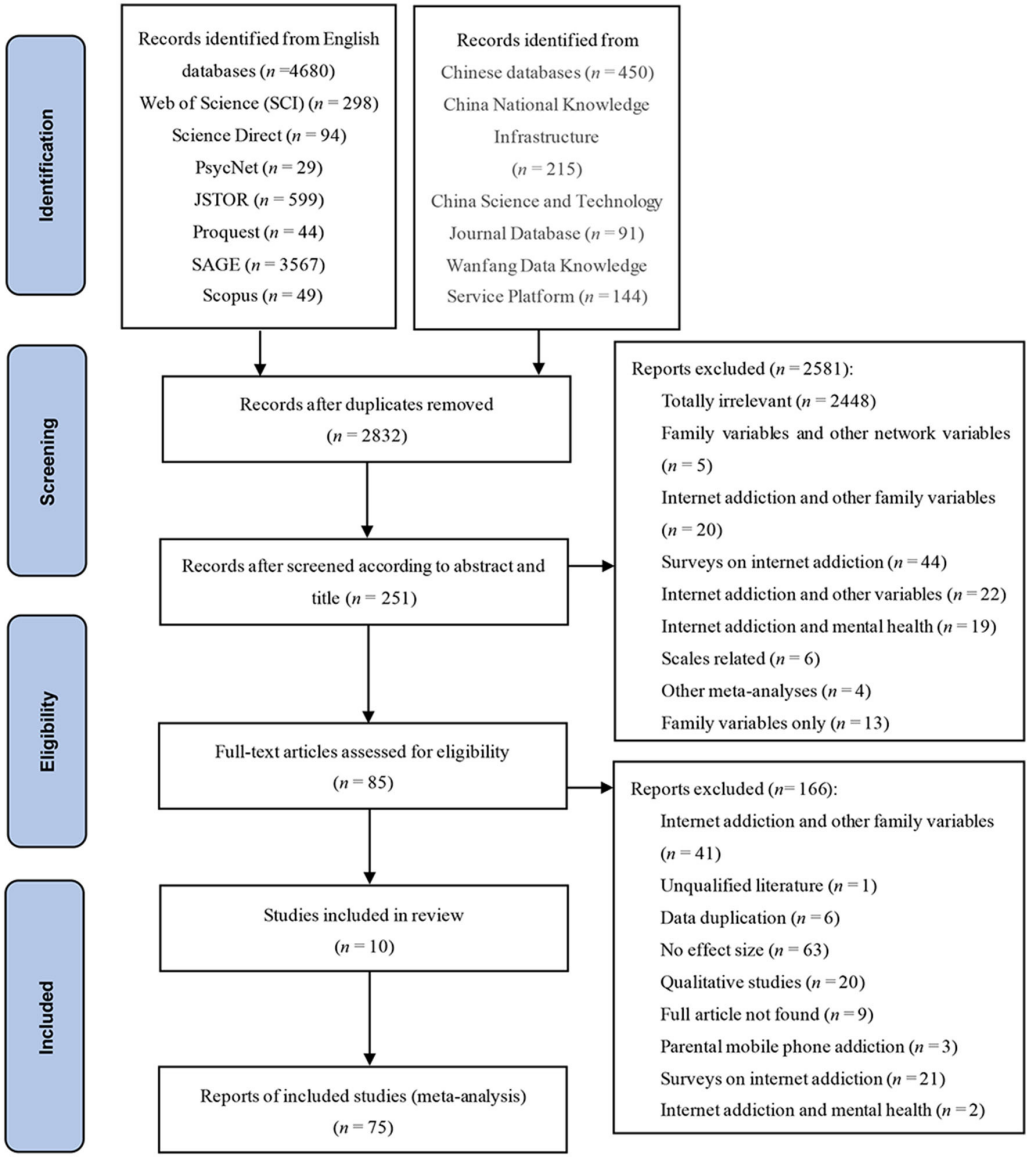
PIRSMA 2009 flow diagram.

This study was conducted under the guidance of the Preferred Reporting Items for Systematic Reviews and Meta-Analyses (PRISMA) (Liberati et al., 2009) (see Figure 1).

There were eight inclusion criteria for the current study: (a) Only cross-sectional studies were included, longitudinal studies were not included; (b) only studies using the scales as a research tool were included; (c) the empirical quantitative studies includes Pearson's product-moment coefficients *r, F*-values, or univariate regression coefficients of the association between parent-child relationship and problematic Internet use, or using the formula to convert non-*r*-values into *r*-values (Card, 2012), (d) studies written in English or Chinese, (e) the children in the parent-child relationships are subjects of studies; (f) self-assessment studies; (g) the quality of the studies met the literature quality scoring criteriaJBI PACES (JBI Critical Appraisal Checklist for Analytical Cross-sectional Studies) (Lin-Lu et al., 2020), this evaluation criterion is applicable to non-experimental and cross-sectional literature, and a score greater than 70% of the total score is considered qualified, we delete an article after review; (h) in order to reduce publication bias, both published(e.g., journal articles, conference papers) and unpublished papers (e.g., master's and doctoral theses) were included. Two exclusion criteria included: Samples' cognitive and judgment abilities were normal, and studies in which samples were psychiatric patients.

Finally, 75 studies meeting the criteria were included, including 34 Chinese studies, 41 English studies, 11 master's and doctoral theses, and 64 journal articles, with 79 independent sample groups and 110,691 participants.

### Code standards

The coding criteria for studies that met the inclusion criteria were as follows: author, year, types of literature, types of problematic Internet use, types of parent-child relationship, age, gender, country, parental identity, scales, and correlation coefficient between parent-child relationship and problematic Internet use (see Table 2). The following points should be noted when coding: (a) Each independent sample was coded as an effect-size value. If multiple scales measure problematic Internet use or parent-child relationship in the same sample, they were also coded as multiple scales to be used as the moderating variables. (b) For studies reporting only the *r*-values of each dimension of the scales, the average value was taken as the effect size for the overall correlation coefficient. For the scales of reverse scoring, *r-*values were converted by sign. (c) The types of parent-child relationship and problematic internet use were coded according to the traits actually measured on the scales. (d) If multiple effect sizes on the strength of the association between parent-child relationship and problematic Internet use in the same sample, only the overall effect size was used. (e) Year is the actual research year to encode.

**Table 2:** Characteristics of the 75 studies included in the meta-analysis.

**Name**	**Year**	**r**	**TL^a^**	**TP^b^**	**TPCR^c^**	**Age^d^**	**%Male**	**Country**	**Object^e^**	**MPCR^f^**	**MPIU^g^**
Akdeniz et al. (2020)	2018	−0.31	1	GA	PCA	2	43.10	Turkey	1	3	3
Badenes-Ribera et al. (2019)	2019	−0.10	1	SMA	PCA	2	45.80	Italy	1	3	6
Bandgi and Suneel (2018)	2017	−0.11	1	GA	PCA	3	39.30	Pakistan	1	7	3
Bilgin et al. (2020) (1)	2018	−0.26	1	SMA	PCR	2	37.40	Turkey	1	5	6
Bilgin et al. (2020) (2)	2018	−0.28	1	SMA	PCR	2	37.40	Turkey	3	5	6
Bilgin et al. (2020) (3)	2018	−0.35	1	SMA	PCR	2	37.40	Turkey	2	5	6
Boniel-Nissim and Sasson (2018) (1)	2017	0.00	1	GA	PCC	2	47.00	Israel	3	7	6
Boniel-Nissim and Sasson (2018) (2)	2017	−0.09	1	GA	PCC	2	47.00	Israel	2	7	6
Boniel-Nissim and Sasson (2018) (3)	2017	0.27	1	GA	PCC	2	47.00	Israel	1	7	6
Chi et al. (2016)	2015	−0.18	1	GA	PCR	3	62.10	China	1	7	3
Faltýnková et al. (2020)	2014	−0.17	1	EIU	PCC	2	51.00	Slovakia	1	7	6
Gao et al. (2020) (1)	2017	−0.36	1	MPA	PCR	1	51.60	China	1	5	5
Gao et al. (2020) (2)	2017	0.30	1	MPA	PCR	2	53.00	China	1	5	5
Gao et al. (2020) (3)	2017	−0.18	1	MPA	PCR	2	53.00	China	1	5	5
Gao et al. (2019)	2017	−0.31	1	MPA	PCR	2	51.80	China	1	5	5
Hong et al. (2019)	2017	−0.24	1	MPA	PCR	2	48.60	China	1	2	5
Hsieh et al. (2018)	2018	−0.19	1	GA	PCR	1	50.30	China	1	7	2
Huang et al. (2019)	2019	−0.36	1	GA	PCR	2	58.30	China	1	7	2
Jeong et al. (2020) (1)	2020	−0.27	1	IGA	PCA	2	53.40	South Korea	3	3	4
Jeong et al. (2020) (2)	2020	−0.38	1	IGA	PCA	2	53.40	South Korea	2	3	4
Kim et al. (2018) (1)	2016	−0.38	1	IGA	PCC	2	55.50	South Korea	3	6	4
Kim et al. (2018) (2)	2016	−0.05	1	IGA	PCC	2	55.50	South Korea	2	6	4
Kim and Kim (2015) (1)	2015	−0.09	1	IGA	PCA	2	51.40	South Korea	3	3	4
Kim and Kim (2015) (2)	2015	−0.15	1	IGA	PCA	2	51.40	South Korea	2	3	4
King and Delfabbro (2016) (1)	2016	−0.18	1	IGA	PCA	2	49.04	Australia	3	3	4
King and Delfabbro (2016) (2)	2016	−0.18	1	IGA	PCA	2	49.04	Australia	2	3	4
Kwon et al. (2009) (1)	2008	0.28	1	IGA	PCF	2	60.92	South Korea	1	7	3
Kwon et al. (2009) (2)	2008	−0.17	1	IGA	PCR	2	60.92	South Korea	1	7	3
Lee and Kim (2018)(1)	2018	−0.28	1	MPA	PCC	2	50.00	South Korea	1	6	6
Lee and Kim (2018)(2)	2018	−0.17	1	MPA	PCR	2	50.00	South Korea	1	6	6
Lepp et al. (2016) (1)	2016	−0.08	1	MPA	PCA	3	20.08	America	1	3	6
Lepp et al. (2016) (2)	2016	−0.15	1	MPA	PCC	3	20.08	America	1	3	6
Liu et al. (2012)	2012	−0.23	1	GA	PCC	2	49.78	China	1	6	1
Liu et al. (2019)	2019	−0.29	1	GA	PCC	2	49.47	China	1	6	1
Malik et al. (2020)	2020	−0.12	1	IGA	PCA	2	52.40	India	1	3	4
Marino et al. (2019) (1)	2019	−0.24	1	EIU	PCA	2	32.10	Italy	2	3	6
Marino et al. (2019) (2)	2019	−0.19	1	EIU	PCA	2	32.10	Italy	3	3	6
Marino et al. (2019) (3)	2019	−0.22	1	EIU	PCC	2	32.10	Italy	2	3	6
Marino et al. (2019) (4)	2019	−0.09	1	EIU	PCC	2	32.10	Italy	3	3	6
Niu et al. (2020)	2020	−0.45	1	MPA	PCR	2	51.45	China	1	4	5
Soh et al. (2018)	2018	−0.21	1	GA	PCA	2	46.60	Malaysia	1	3	6
Qiao and Liu (2020)	2020	−0.24	1	MPA	PCR	2	45.70	China	1	7	6
Liu et al. (2013) (1)	2013	−0.21	1	GA	PCR	2	48.50	China	3	1	1
Liu et al. (2013) (2)	2013	−0.20	1	GA	PCR	2	48.50	China	2	1	1
Zhen et al. (2019)	2017	−0.21	1	MPA	PCR	2	50.23	China	1	7	6
Say and Batigun (2016) (1)	2016	−0.10	2	GA	PCI	3	39.60	Turkey	3	4	6
Say and Batigun (2016) (2)	2016	−0.12	2	GA	PCI	3	39.60	Turkey	2	4	6
Shek et al. (2019) (1)	2013	−0.14	1	GA	PCR	2	51.30	China	3	7	3
Shek et al. (2019) (2)	2013	−0.13	1	GA	PCR	2	51.30	China	2	7	3
Shek et al., 2018 (1)	2010	−0.27	1	GA	PCR	2	52.10	China	3	7	3
Shek et al. (2018) (2)	2010	−0.24	1	GA	PCR	2	52.10	China	2	7	3
Soh et al. (2014)	2014	−0.20	1	GA	PCA	2	46.80	Malaysia	1	3	6
Teng et al. (2020) (1)	2017	−0.16	1	IGA	PCA	3	41.20	China	2	3	4
Teng et al. (2020) (2)	2018	−0.24	1	IGA	PCA	3	36.90	China	2	3	4
Teng et al. (2020) (3)	2018	−0.29	1	IGA	PCA	3	38.00	China	2	3	4
Teng et al. (2020) (4)	2017	−0.15	1	IGA	PCA	3	41.20	China	3	3	4
Teng et al. (2020) (5)	2018	−0.22	1	IGA	PCA	3	36.90	China	3	3	4
Teng et al. (2020) (6)	2018	−0.34	1	IGA	PCA	3	38.00	China	3	3	4
Trumello et al. (2018) (1)	2017	−0.11	1	GA	PCI	2	42.40	Italy	3	7	6
Trumello et al. (2018) (2)	2017	−0.05	1	GA	PCI	2	42.40	Italy	2	7	6
Wang et al. (2018)	2017	−0.25	1	GA	PCR	2	47.00	China	1	4	3
Wei et al. (2020)	2019	−0.35	1	GA	PCA	2	53.81	China	1	3	3
Li and Hao (2019)	2019	−0.29	1	MPA	PCA	2	50.79	China	1	3	5
Xie et al. (2019)	2015	−0.36	1	MPA	PCA	2	48.56	China	1	3	6
Yang et al. (2016) (1)	2015	−0.14	1	GA	PCA	3	38.10	China	3	3	2
Yang et al. (2016) (2)	2015	−0.20	1	GA	PCA	3	38.10	China	2	3	2
Zhang et al. (2020)	2019	−0.25	1	MPA	PCA	3	41.00	China	1	3	5
Zhu et al. (2015)	2013	−0.11	1	IGA	PCR	2	52.17	China	1	5	3
Chen et al. (2016) (1)	2014	−0.35	1	GA	PCC	3	49.12	China	3	6	2
Chen et al. (2016) (2)	2014	−0.34	1	GA	PCC	3	49.12	China	2	6	2
Chen et al. (2018) (1)	2018	−0.19	1	GA	PCA	2	45.69	China	2	3	3
Chen et al. (2018) (2)	2018	−0.18	1	GA	PCA	2	45.69	China	2	3	3
Chen et al. (2019) (1)	2017	−0.20	1	EIU	PCI	2	53.51	China	3	2	6
Chen et al. (2019) (2)	2017	−0.17	1	EIU	PCI	2	53.51	China	2	2	6
Deng et al. (2013b) (1)	2013	−0.20	1	GA	PCA	2	46.24	China	3	3	2
Deng et al. (2013b) (2)	2013	−0.22	1	GA	PCA	2	46.24	China	2	3	2
Deng et al. (2014) (1)	2014	−0.22	1	GA	PCC	2	45.71	China	3	6	2
Deng et al. (2014) (2)	2014	−0.22	1	GA	PCC	2	45.71	China	2	6	2
Ding et al. (2020) (1)	2019	−0.11	1	MPA	PCA	3	17.00	China	3	3	5
Ding et al. (2020) (2)	2019	−0.03	1	MPA	PCA	3	17.00	China	3	3	5
Feng et al. (2017) (1)	2015	−0.28	1	GA	PCC	3	26.30	China	3	6	2
Feng et al. (2017) (2)	2015	−0.29	1	GA	PCC	3	26.30	China	2	6	2
Gao et al. (2009)	2007	−0.14	1	GA	PCR	2	53.75	China	1	7	2
Guo et al. (2018) (1)	2018	−0.07	1	GA	PCI	1	53.64	China	3	4	3
Guo et al. (2018) (2)	2018	0.20	1	GA	PCF	1	53.64	China	3	4	3
Guo et al. (2018) (3)	2018	−0.08	1	GA	PCI	1	53.64	China	2	4	3
Guo et al. (2018) (4)	2018	0.14	1	GA	PCF	1	53.64	China	2	4	3
Huang (2019) (1)	2019	−0.22	2	GA	PCA	2	69.17	China	3	3	1
Huang (2019) (2)	2019	−0.24	2	GA	PCA	2	69.17	China	2	3	1
Liu (2017)	2017	0.11	1	GA	PCR	2	50.69	China	1	7	2
Liu and Luo (2010)	2010	−0.27	1	GA	PCI	2	51.20	China	1	2	2
Lv (2019) (1)	2019	−0.26	2	GA	PCA	2	42.90	China	3	3	1
Lv (2019) (2)	2019	−0.22	2	GA	PCA	2	42.90	China	2	3	1
Mu (2017)	2017	−0.11	1	MPA	PCA	3	48.89	China	1	3	5
Qian (2019)	2019	−0.12	2	MPA	PCA	2	49.41	China	1	3	5
Qing et al. (2017) (1)	2017	−0.17	1	MPA	PCA	3	23.63	China	3	3	6
Qing et al. (2017) (2)	2017	−0.16	1	MPA	PCA	3	23.63	China	2	3	6
Shi (2020)	2020	0.19	2	IGA	PCF	2	44.48	China	1	7	6
Su et al. (2016)	2012	−0.14	1	IGA	PCR	2	48.90	China	1	5	3
Tian et al. (2017) (1)	2017	−0.23	1	IGA	PCR	2	49.96	China	3	5	6
Tian et al. (2017) (2)	2017	−0.19	1	IGA	PCR	2	49.96	China	2	5	6
Wei (2007) (1)	2007	0.21	2	GA	PCF	2	50.33	China	1	7	2
Wei (2007) (2)	2007	−0.18	2	GA	PCI	2	50.33	China	1	2	2
Wu (2013)	2013	−0.20	2	GA	PCR	2	62.20	China	1	7	3
Wu (2007) (1)	2007	−0.30	2	GA	PCA	2	52.80	China	3	3	1
Wu (2007) (2)	2007	−0.11	2	GA	PCA	2	52.80	China	2	3	1
Xie and Ding (2018)	2018	−0.34	1	SMA	PCC	3	31.70	China	1	6	6
Xu (2009)	2009	−0.42	1	GA	PCC	3	30.10	China	1	6	3
Yang (2021)	2019	−0.25	1	GA	PCC	2	58.92	China	1	6	6
Yu (2018)	2018	−0.13	2	MPA	PCR	2	54.14	China	1	1	6
Zhang et al. (2011) (1)	2011	−0.18	1	GA	PCR	2	49.16	China	3	1	1
Zhang et al. (2011) (2)	2011	−0.17	1	GA	PCR	2	49.16	China	2	1	1
Zhang (2019) (1)	2019	−0.18	2	EIU	PCR	2	47.90	China	3	2	6
Zhang (2019) (2)	2019	−0.10	2	EIU	PCR	2	47.90	China	2	2	6
Zhang and Zhang (2018) (1)	2017	−0.31	1	GA	PCC	3	46.95	China	3	6	2
Zhang and Zhang (2018) (2)	2017	−0.30	1	GA	PCC	3	46.95	China	2	6	2
Zhang et al. (2015) (1)	2013	−0.20	1	IGA	PCR	2	51.06	China	3	5	4
Zhang et al. (2015) (2)	2013	−0.18	1	IGA	PCR	2	51.06	China	2	5	4
Zhang et al. (2018)	2016	−0.25	1	MPA	PCA	3	40.44	China	1	3	5
Zhao et al. (2018) (1)	2017	−0.29	1	GA	PCA	2	47.40	China	1	3	3
Zhao et al. (2018) (2)	2017	0.31	1	GA	PCA	2	47.40	China	1	7	3
Zheng (2015) (1)	2015	0.28	2	EIU	PCI	2	43.80	China	1	7	6
Zheng (2015) (2)	2015	−0.06	2	EIU	PCF	2	43.80	China	1	7	6
Zhou (2020)	2020	0.35	2	IGA	PCC	2	49.30	China	1	6	4

^a^TL, Types of Literature; 1 = journal article, 2 = thesis; ^b^TP, Types of Problematic Internet Use; EIU, excessive Internet use; GA, general addiction; IGA, internet game addiction; MPA, mobile phone addiction; SMA, social media addiction. ^c^TPCR, Types of Parent-child relationship; PCA, parent-child attachment; PCC, parent-child communication; PCF, parent-child conflict; PCI, parent-child affinity; PCR, parent-child relationship. ^d^Age: 1 = children, 2 = adolescents, 3 = emerging adult. ^e^Object = Object of parent-child relationship: 1= parents, 2 = mother, 3 = father. ^f^MPCR, measures of Parent-child relationship; 1 = Closeness to Parents Scale (CPS), 2 = Cohesion Evaluation Scales (FACES), 3 = Inventory of Parent and Peer Attachment (IPPA), 4 = Parent-Adolescent Child Relationship Questionnaire (PACRQ), 5 = Parent-Child Relationship Questionnaire (PARQ), 6 = Parent-Child Communication Scale (PCCS), 7 = Others. ^g^MPIU, measures of Problematic Internet use; 1 = Adolescent Pathological Internet Use Scale (APIUS), 2 = Chen Internet Addiction Scale (CIAS), 3 = Internet Addiction Test (IAT), 4 = Internet Gaming Disorder Scale (IGDS), 5 = Mobile Phone Problem Use Scale (MPPUS), 6 = Others.

The coding standards of the regulating variables are as follows. According to the severity of problematic Internet use (Davis, 2001; Su and Lin, 2014; Su et al., 2014) and the cognitive-behavior model (Davis, 2001), which divides problematic Internet use into “generalized problematic Internet use” and “special types of problematic Internet use”. Special types of problematic Internet use refer to people who use the Internet for specific purposes (e.g., SMD, IGD). Therefore, problematic Internet use can be divided into the following five types: general internet addiction(GIA) (generalized Internet addiction, do not distinguish the types of problematic Internet use) (Ho et al., 2014), social media addiction(SMA) (uncontrollable and abnormal use of social media, which meets the addiction standards), excessive Internet use(EIU) (excessive use of Internet, but not meeting addiction standards), mobile phone addiction(MPA) (uncontrollable and abnormal use of mobile phone, which meets the addiction standards), and Internet game addiction(IGA) (excessive and abnormal use of online games, which meets the addiction standards). However, researchers did not exactly distinguish among these concepts, so the classification criteria will be determined based on the problematic Internet use measured by the scales. The parent-child relationship types were coded as “parent-child relationship”, “parent-child attachment”, “parent-child communication” and “parent-child affinity”, “parent-child conflict” according to the actually measured type of the scales.

We also tested differences in the association between the quality of parent-child relationship and problematic Internet use across countries. Because different scales have different standards, most of the measured characteristics coincide with each other. Therefore, multiple versions of the same scale are coded as one type. Only the scales used more were coded, and the scales used less were coded as other types of scales. Parental Identity in the Parent-child Relationship was coded as the father, the mother and the parents. Gender was coded according to the sex ratio of boys recorded in the studies. Age groups were coded as children (012 years old), adolescents (12−17 years old) (Lin and Li, 2004), and emerging adults (18−25 years old) (Arnett, 2000). In addition, we test for differences among literature types. The studies included in this meta-analysis spanned nearly two decades, and we also tested differences across years.

Our coders coded at different times according to the coding standards, and its consistency was 96%, and any inconsistencies were corrected according to the coding standards.

### Analysis process

In this study, Comprehensive Meta-Analysis (version 3.0)^1^ was used to take the *r*-values of the association between parent-child relationship and problematic Internet use as the effect sizes. We use the Fisher's *z*-test to transform *r*-values into *Z*-values for analysis. During the conversion process, weights were inserted according to the size of each sample. The Z-scores were transformed into the mean value and confidence interval of the correlation coefficients (Lipsey and Wilson, 2001).

First, publication bias was assessed to avoid incomplete literature retrieval. In order to avoid incomplete literature retrieval, both unpublished (master's and doctoral theses) and published (journal papers, conference papers) studies were included. In addition, the funnel plots, *classic fail-safe N* and *Egger's test* (Egger et al., 1997) were used to test publication bias. Suppose the funnel plots have a symmetric distribution on both sides. In that case, the *classic fail-safe N* is significant and the *Egger's test* is non-significant, the possibility of publication bias is very low.

Second, a heterogeneity test was conducted to test whether the effect sizes were heterogeneous. A random effect model was used if heterogeneous (*p* ≤ 0.05); otherwise, a fixed effect model was used. Furthermore, if the *Q* test was significant and *I*^2^ > 75, it indicates there are heterogeneous among effect sizes, then main effect analysis and moderating effect analysis were analyzed (Higgins et al., 2002).

Finally, the main effect and the moderating effect were analyzed. The moderating effect included meta-regression analysis and subgroup analysis. The meta-regression analysis was for the continuous moderating variable, and the meta-subgroup analysis was for the categorical moderating variables. In subgroup analysis, the effect sizes of each dimension should not be less than five, and the dimensions with less than five effect sizes should not be calculated (Card, 2012).

## Results

### Homogeneity test

The results of the heterogeneity test performed on the effect sizes in this study showed that the included effect sizes were heterogeneous [*Q*_(123)_ = 3017.16, *I*^2^ = 95.92, *p* < 0.001] and *I*^2^ 75, indicating a high degree of heterogeneity among the effect sizes. Hence, the random effect model was selected for the meta-analysis.

### Main effects

The random effect model was used to test the strength of the association between parent-child relationship and problematic Internet use, and a significant negative correlation between the two was found (*r* = −0.18, 95% *CI* = [−0.20, −0.15], *Z* = −14.35, *Q*_(123)_ = 3017.16, *p* < 0.001). According to the criteria proposed for quantitative analysis, 0.1, 0.2 and 0.3 are regarded as small-level, middle-level, and large-level correlation coefficients, respectively (Gignac and Szodorai, 2016). Hence, the parent-child relationship was middle-level correlated with problematic Internet use.

**Figure 2:**
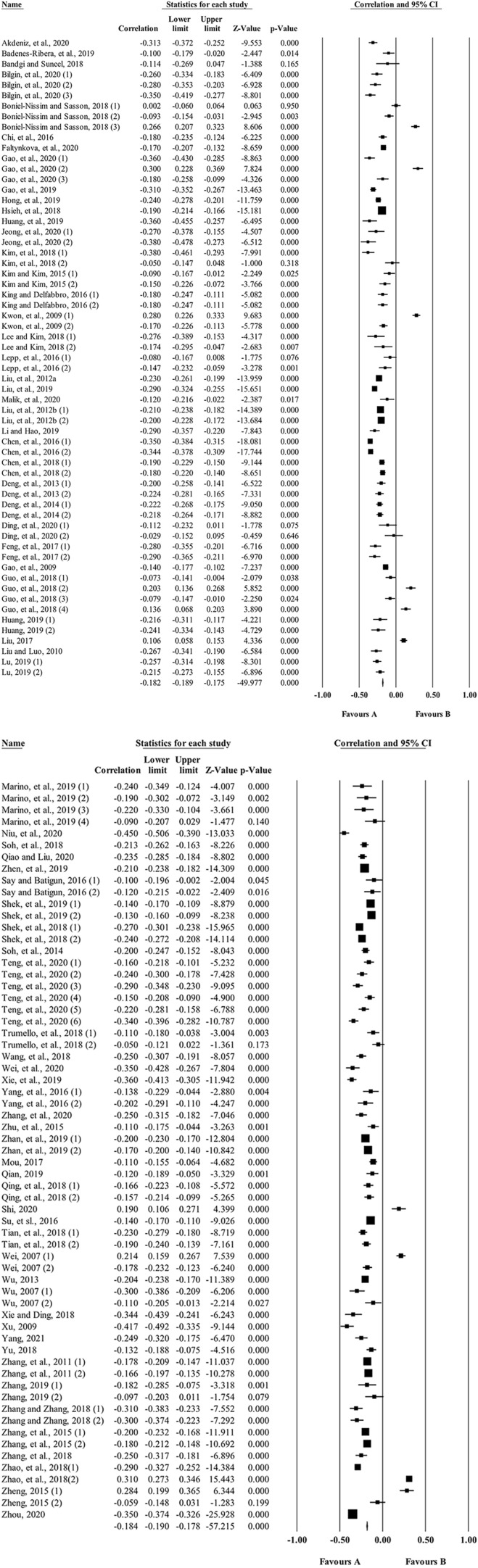
Forest plot for the link between parent-child relationship and PIU.

The forest plots present the results of all studies (95% confidence intervals used for standardized differences of the means). See Figure 2.

### Publication bias

**Figure 3:**
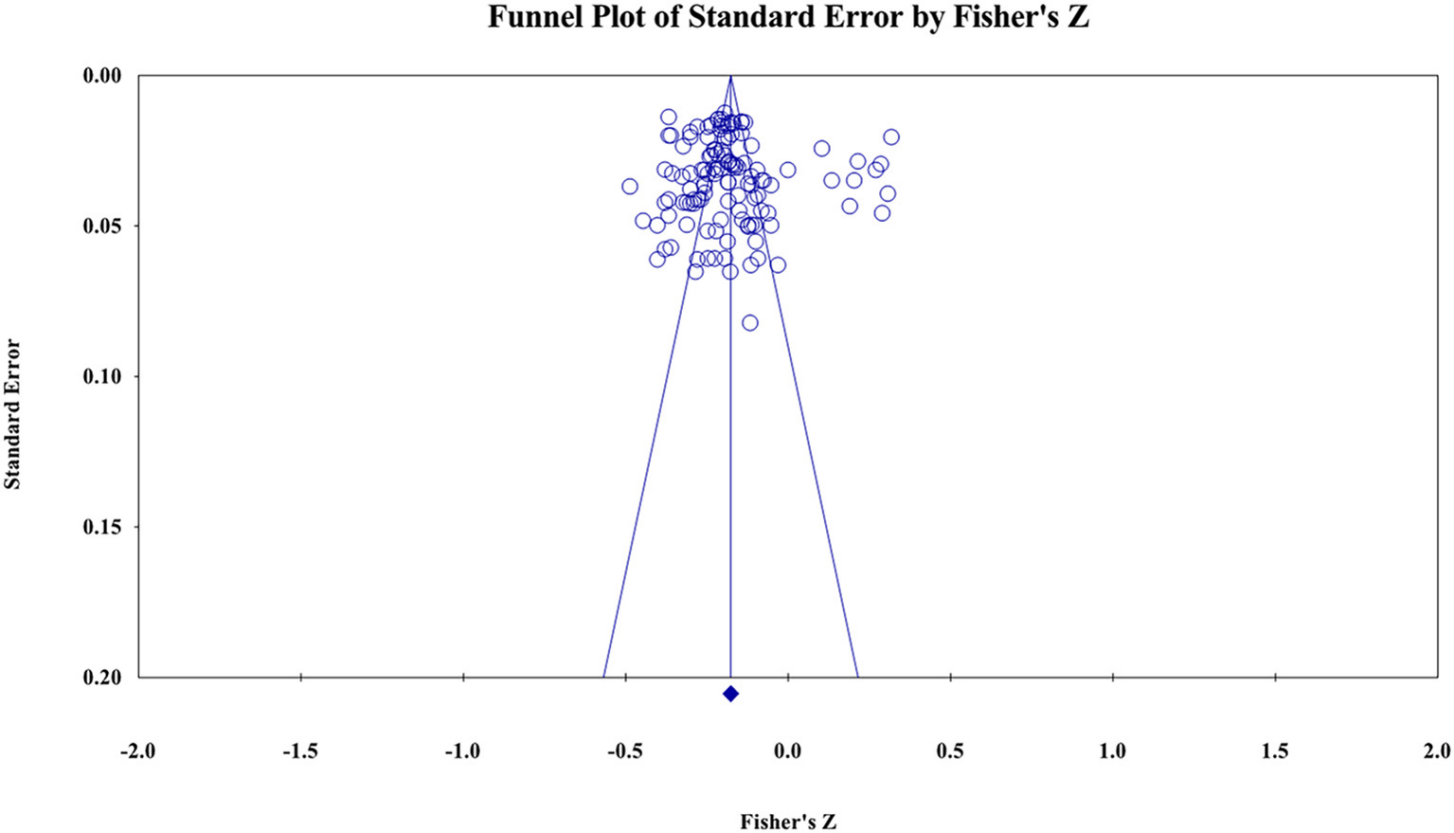
Funnel plot of effect sizes of the correlation between parent-child relationship and PIU.

The funnel plot (see Figure 3) shows that the effect values have a symmetric distribution around the mean value, and the probability of publication bias is low. The results of *Classic fail-safe N* showed *Z* = −67.11, *p* < 0.05 and *Egger's test* showed *t* = 1.09, *p* > 0.05. Therefore, the possibility of publication bias in this study was extremely low.

### Moderator analysis

Subgroup analyses and meta-regression were used to test whether the strength of the association between parent-child relationships and problematic Internet use was influenced by the moderate indicators.

#### Subgroup analyses

Subgroup analysis was conducted on the moderating variables of types of literature publication, types of problematic Internet use, types of parent-child relationship, age, country, parental identity and scales.

a) The homogeneity test found that the types of problematic Internet use did not moderate the negative link between parent-child relationship and problematic Internet use [*Q* (4) = 7.02, *p* > 0.05]. Further homogeneity test found that the association between SMA (*r* = −0.27) and the parent-child relationship was significantly stronger than the association between EIU (*r* = −0.12) and the parent-child relationship [*Q* (1) = 6.47, *p* < 0.05] (see Table 3).
b) The types of parent-child relationship significantly moderate the link between parent-child relationship and problematic Internet use [*Q* (4) = 75.67, *p* < 0.001]. However, the further homogeneity test indicated that the association between parent-child communication (*r* = −0.23) and problematic Internet use was stronger than the association between parent-child affinity (*r* = −0.10) and problematic Internet use [*Q* (1) = 8.44, *p* < 0.01] (see Table 3).
c) Age did not moderate the association between parent-child relationship and problematic Internet use. But the association between parent-child relationship and problematic Internet use in emerging adults (*r* = −0.22) was stronger than in adolescents (*r* = −0.17) [*Q* (1) = 4.55, *p* < 0.05] (see Table 3).
d) Country did not moderate the association between parent-child relationship and problematic Internet use [*Q* (3) = 5.99, *p* > 0.05, see Table 3]. Specifically, the association between the parent-child relationship and problematic Internet use in Italy (*r* = −0.13) was significantly weaker than in Turkey (*r* = −0.24) [*Q* (1) = 5.46, *p* < 0.05] or approached significantly weaker in China (*r* = −0.19) [*Q* (1) = 3.07, *p* = 0.08 < 0.01].
e) Parental identity of parent-child relationship did not moderate the association between the parent-child relationship and problematic Internet use [*Q* (2) = 1.11, *p* > 0.05] (see Table 3).
f) The parent-child relationship scales significantly moderated the association between the parent-child relationship and problematic Internet use [*Q* (4) = 25.65, *p* < 0.001, see Table 3]. Specifically, the association between the parent-child relationship and problematic Internet use was significantly stronger using the PCCS (*r* = −0.29) than using the CPS (*r* = −0.18) [*Q* (1) = 24.65, *p* < 0.001], the IPPA (*r* = −0.21) [*Q* (1) = 15.38, *p* < 0.001], the FACES (*r* = −0.20) [*Q* (1) = 14.81, *p* < 0.001], or the PARQ (*r* = −0.19) [*Q* (1) = 6.38, *p* < 0.05].
g) The problematic Internet use measures significantly moderate the association between parent-child relationship and problematic Internet use [*Q* (5) = 13.53, *p* < 0.05, see Table 3]. However, the further homogeneity test indicated that association between parent-child relationship and problematic Internet use using the IAT (*r* = −0.13) was significantly weaker than using the IGDS (*r* = −0.22) [*Q* (1) = 4.70, *p* < 0.05] or the APIUS (*r* = −0.22) [*Q* (1) = 5.48, *p* < 0.05]. The association between parent-child relationship and problematic Internet use using the IGDS (*r* = −0.22) was significantly stronger than using the other scales (*r* = −0.15) [*Q* (1) = 5.81, *p* < 0.05].

**Table 3:** Moderator analysis of correlations between parent-child relationship and problematic Internet use.

**Moderate variable**	**Between-group effect (Q*_*BET*_*)**	**Category**	**k**	**Mean *r***	**SE**	**95% CI for** ***r***		**Test of null (two-tailed) z-value within each group (Q_W_)**
						**LL**	**UL**	
Types of problematic Internet use	6.47*	EIU	11	−0.12	0.01	−0.19	−0.07	−3.62***
		SMA	5	−0.27	0.01	−0.35	−0.18	−5.58***
Types of parent-child relationships	8.44**	PCI	11	−0.10	0.01	−0.16	−0.04	−3.04**
		PCC	24	−0.23	0.01	−0.28	−0.17	−7.73***
Age	4.55*	EA	29	−0.22	0.01	−0.26	−0.18	−11.43***
		Ado	89	−0.17	0.02	−0.20	−0.14	−11.60***
Countries	5.96^+^	Turkey	6	−0.24	0.00	−0.32	−0.17	−6.02***
		Italy	7	−0.13	0.00	−0.18	−0.08	−4.96***
		China	89	−0.19	0.00	−0.21	−0.16	−13.40***
Object of Parent-child relationship	1.11	Parents	54	−0.16	0.01	−0.21	−0.11	−6.60***
		Father	35	−0.19	0.00	−0.22	−0.16	−11.20***
		Mother	35	−0.19	0.00	−0.21	−0.16	−13.32***
Parent-child relationship measures	25.65***	CPS	5	−0.18	0.00	−0.21	−0.16	−15.76***
		FACES	7	−0.20	0.00	−0.23	−0.17	−12.41***
		IPPA	47	−0.21	0.01	−0.23	−0.18	−18.19***
		PCCS	18	−0.29	0.01	−0.16	−0.13	−15.87***
		PCRQ	13	−0.19	0.01	−0.26	−0.13	−5.77***
Problematic Internet use measures	13.53***	APIUS	12	−0.22	0.00	−0.24	−0.19	−15.92***
		IGDS	18	−0.22	0.01	−0.26	−0.18	−9.42***
		MPPUS	13	−0.19	0.03	−0.28	−0.10	−4.00***
		IAT	23	−0.13	0.29	−0.20	−0.06	−3.56***
		CIAS	19	−0.21	0.02	−0.27	−0.14	−6.03***
		Others	39	−0.15	0.01	−0.19	−0.11	−7.41***

^+^ *p* < 0.1, *p < 0.05, **p < 0.01, ***p < 0.001.

#### Meta-regression analysis

Meta-regression analysis of year and gender found that (see Table 4) year (*Q*_Model_ [1, *k* = 124] = 1.46, *p* > 0.05) and gender (Q_Model_ [1, k = 124] = 0.48, *p* > 0.05) did not significantly moderate the association between parent-child relationship and problematic Internet use.

**Table 4:** Meta-regression analyses of gender and year.

**Variable**	**Parameter**	**Estimate**	***SE***	***z*-value**	**95% CI for** ***b***	
					**LL**	**UL**
Male (%)	β_0_	−0.220	0.063	−3.50	−0.34	−0.10
	β_1_	0.001	0.001	0.69	−0.002	0.004
	Q_Model_ (1, k = 124) = 0.48, *p* > 0.05
Year	β_0_	9.08	7.66	1.19	−5.93	−24.10
	β_1_	−0.005	0.004	−1.21	−0.01	−0.003
	Q_Model_ (1, k = 124) = 1.46, *p* > 0.05

## Discussions

### The association between the parent-child relationship and problematic internet use

These results showed that the quality of parent-child relationship was negatively associated with the problematic Internet use, which is consistent with the results of previous studies (Deng et al., 2013a; Lee et al., 2016). Poor interpersonal relationship aggravates problematic Internet use; problematic Internet use also have a negative impact on interpersonal relationship, showing a dynamic two-way interactive relationship (Pardini et al., 2008). According to system theory (Johnson and Ray, 2016), the use of Internet technology will lead to changes in the family system. Family structure theory (James and MacKinnon, 1986) emphasizes the importance of roles and boundaries for family members. The introduction of family communication technology may conflict with the roles and boundaries of the family, resulting in problems in the parent-child system (Tadpatrikar et al., 2021). Besides, from the viewpoint of the information process, the mind sponge mechanism assumes that parents and Internet can be deemed as two main information sources for children. Information-seeking behaviors are driven by the information availability/accessibility and perceived benefits of the information (Nguyen et al., 2021). Therefore, if the parent-child relationship is not good, the children will perceive seeking information from their parents as costly and tend to seek information from other available sources of information, including the Internet, to meet their demands. The demands of children can be various, ranging from problem-solving to entertainment (Vuong, 2022). If this phenomenon persists, it can lead to problematic Internet use.

Hence, attention should be paid to the impact of the Internet and other new technologies on the family system. In making full use of the Internet, we should clarify the boundaries among family subsystems. Furthermore, new rules should be formulated to make the Internet use become a lubricant among family members. However, the results of a meta-analysis showed that problematic Internet use was influenced by both personal and social factors (Fumero et al., 2018). Compared with social factors, personal factors were more strongly associated with problematic Internet use, and the relationship between emotional problems in personal factors and Internet addiction was stronger. Another meta-analysis investigated the association between loneliness (personal factors) and problematic Internet use and found that the two showed a middle-level correlation between loneliness and problematic Internet use (*r* = 0.25) (Zhang et al., 2014) in this study, indicating that the strength of the association between loneliness and problematic Internet use (*r* = 0.18) was stronger than that of in this study (Fumero et al., 2018). Therefore, future research on problematic Internet use needs to combine individual and social factors for intervention and exploration.

### The moderating effects of the association between the parent-child relationship and problematic internet use

#### Types of problematic internet use

Compared with EIU, the association between SMA and the quality of parent-child relationship is stronger. The Internet has become an important and powerful social tool in daily life (Badenes-Ribera et al., 2019; Bilgin et al., 2020; Demircoglu and Kose, 2020), and the main reason why most people use social media is to seek socialization (Bilgin et al., 2020). Social media addicts unable to control social media use, which interferes with their normal life (Ryan et al., 2014). According to attachment theory, insecure parent-child attachment will lead individuals to seek social support (Jenkins-Guarnieri et al., 2012; Monacis et al., 2017) and establish connections with the outside world, and social media provides an opportunity to seek social attachment. Social support theory (Cutrona et al., 1994) also emphasizes the importance of social support for individuals. People who are frustrated in real life often seek support in the virtual world. The Internet use disorder model (Ceyhan et al., 2018) suggests that problematic Internet use is bidirectionally associated with loneliness (Zhen et al., 2019), low self-esteem (Demircoglu and Kose, 2020) and other factors related to cognition, emotion and executive function. The poor parent-child relationship can lead to these problems, thus exacerbating the problematic Internet users to escape from reality and relieve pressure through the Internet. Social communication with friends on the Internet and rumination (“only the Internet accompanies them”) further reduce their desire to communicate with their parents and further reduce the quality of the parent-child relationship (Davis, 2001).

Compared with other types of problematic Internet use, social media addiction may be a particular form (Davis, 2001), emphasizing social support and attachment. Its online behavior has a more specific purpose and is more likely to cause symptoms (such as withdrawal). Therefore, social media addiction is more closely related to the parent-child relationship. However, the EIU in this study did not reach the level of behavioral addiction (Zhang, 2019), indicating that the desire for and dependence on the Internet (Younes et al., 2016) affects normal life. It may indicate that the deterioration of the parent-child relationship may lead to EIU and even cause them to become internet addicts. We should pay attention to the influence of parental support on children, satisfy children's needs to receive support from parents in real life, prevent them from seeking support in the Internet world, and reduce their possibility of problematic Internet use. In the future, more attention should be given to the positive significance of social media for training social skills (D'Arienzo et al., 2019).

#### Participant characteristic

This study found that the association between the parent-child relationship and problematic Internet use in emerging adults was significantly stronger than that in adolescents, and most college students were in the adult emerging stage. Previous studies generally considered that adolescents are in a period of agitation with an immature prefrontal lobe, and are more likely to engage in impulsive behaviors (Kim et al., 2018), such as IA (Chi et al., 2016). And emphasizing the correlation between problematic Internet use (Lyu, 2017; Ballarotto et al., 2018; Awaluddin et al., 2019; Badenes-Ribera et al., 2019) and parent-child relationship among adolescents, few studies have focused on IA of college students in adult emerging stage (Chi et al., 2016). College students have more free time, and some studies have found that the prevalence of IA is higher among college students (Liu et al., 2011). The availability of the Internet makes them intolerant to pressure, pain, and lack of psychological support and more likely to access the Internet to relieve pain (Akbari, 2017). Emerging adults have greater psychological and social development needs than middle and high school students. Moreover, emerging adults who leave their families need to keep in touch with their families through the Internet (Lepp et al., 2016), while the poor parent-child relationship may increase the possibility of problematic Internet use (Chi et al., 2016). What's more, the mind sponge mechanism assumes that a person's thinking and behaviors are driven by his/her value system shaped by the mindset (or a set of core values) (Vuong and Napier, 2015). Such behaviors also include information-seeking behaviors. People from different cultures and at different ages have different mindsets.

The results of this study suggest that we should pay more attention to the problematic Internet use of emerging adults. Emerging adults are a transition stage for individuals to become adults, and good social support is vital for them. Moreover, the Internet is an essential means in their life and study. They study, socialize, and consult information through the Internet. A number of studies have shown that emerging adults spend an average of more than 5 h on the Internet daily (Lepp et al., 2016). Rational use of the Internet can benefit their lives, while excessive Internet use further hinders their adaptation to society. Therefore, it is necessary to regulate emerging adults' use of the Internet and provide them with social support to help them get through this stage smoothly.

#### Country

The association between the parent-child relationship and problematic Internet use was weaker in Italy than in Turkey or China. Cultural differences between countries lead to differences in parenting styles. According to Hofstede's six-dimensional model of national cultural dimension model (Liao, 2014), on a scale of 100, the results (Burton et al., 2021) show that China (80 points) and Turkey (66 points) have higher power distance scores than Italy (50 points), and the society is more hierarchical, and individuals in the society are more accepting of this kind of hierarchical difference. China and Turkey emphasize the awareness of rules, and parents have strong constraints on their children and tend to adopt authoritarian parenting styles. The younger generation communicates in a compromising, non-confrontational way, with certain crises and chaos behind it. In such a complex family model, parent-child communication is blocked (Liu et al., 2019), the parent-child relationship is tense, and children have weak self-restraint, making them more prone to problematic Internet use. However, in Italian, individuals are more independent and care more about their feelings, and the problematic Internet use is less affected by parent-child relationships.

Second, Italy is much more individualistic (76 points) than China (20 points) and Turkey (37 points), and Italians are less connected to their families than those in China and Turkey. Countries with higher levels of individualism have higher subjective wellbeing; they will be happy if they are different from the group. Italian parents cultivate their independence, encourage their children to express themselves, and use authoritative education methods (Hillekens et al., 2020). However, in collectivist countries, such as China, parents tend to cultivate their children to integrate into the collective, suppress their self-expression, and adopt authoritarian education methods. Moreover, they care more about their own group impression and follow the advice of authority figures (such as parents). If they are more integrated with their family, they will feel happy; otherwise, they will have more negative emotions, such as depression (Burton et al., 2021). Negative emotions can further lead to problematic Internet use (Young et al., 2011; Lim, 2012; Cheung et al., 2014; Zhen et al., 2019).

In addition, this study verifies the cultural value model (Ho et al., 2008). The pressure of education competition in China and Turkey is great. Students have nowhere to vent their learning pressure and often seek happiness and support from the Internet. Especially in China, a sense of family collective honor is emphasized (Leung, 2021). Parents will strive to ensure their children's academic performance, arranging multiple cram schools, which is especially marked by excessive parenting. Therefore, it can be found that parent-child intimacy has a more significant protective effect on problematic Internet use under authoritarian parenting style, suggesting that individuals should pay attention to the protective effect of parent-child relationship in an environment that values power. A meta-analysis indicated that countries in the Middle East had higher rates of problematic Internet use than countries in Oceania and Northern and Western Europe. Researchers found that countries with lower life satisfaction, higher environmental pollution, higher commuting time, and lower per capita GDP had a higher prevalence of problematic Internet use (Cheng and Li, 2014). In 2020 (Programme, 2020; Fund, 2021), Italy's Human Development Index (HDI) score was 0.89 (out of 1), and its per capita GDP was $31,300. China's HDI was 0.761, and its per capita GDP was $10,500. Turkey's HDI was 0.820, and its per capita GDP was $8,548. Human Development Index (HDI) was proposed by the United Nations (Programme, 2020), which consists of human life expectancy, years of education and per capita gross national income. The higher the socioeconomic status of the family is, the better the parent-child relationship is, and the lower the possibility of children and adolescents having problematic Internet use (Dong et al., 2019).

Future research can further clarify the influence mechanism of economic, cultural and political factors in different countries on the relationship between parent-child relationship and problematic Internet use.

#### Measures

The association between parent-child relationship and problematic Internet use was significantly stronger using the PCCS than using the FACES, CPS, IPPA, or PARQ. The PCCS is a scale used to measure parent-child communication. Furthermore, the link between parent-child communication and problematic Internet use was stronger than the link between parent-child affinity and problematic Internet use. As mentioned above, most people use the Internet primarily for communication, but the functions of online and offline social networking seem to be different. Compensation network use theory (Karaaslan and Mahoney, 2015) states that online communication will reduce the scope and quality of offline communication (Kraut et al., 1998), while unsatisfied offline communication will aggravate online communication. The results of this study suggest the importance of parent-child communication in preventing problematic Internet use. Some studies have further investigated the relationship between parent-child communication and problematic Internet use, emphasizing the moderating effect of parental reaction on this relationship, and a positive parental response to parent-child communication can further reduce the prevalence of problematic Internet use (Liu et al., 2019). Hence, interventions to strengthen parent-child communication are more direct and effective approaches to prevent problematic Internet use.

Second, the association between the parent-child relationship and problematic Internet use using the IAT was significantly weaker than the IGDS or the APIUS. The IAT is mainly used to assess IA, but there is no unified dimension division. Since it is adapted from the criteria of pathological gambling in DSM-5, self-assessment may affect its accuracy (Su and Lin, 2014), so it should be used cautiously. The APIUS is mainly used to measure PIU, which is a questionnaire compiled by selecting the core dimensions based on the diagnostic criteria for impulse control disorders in DSM-5 and the dimension definition of PIU in existing journals (Lei and Yang, 2007). Some researchers declared that the focus of PIU is different from that of IA, which is more emphasized as a disease and has a more precise standard division. IA is more emphasized as a disease with a clearer standard division (Su et al., 2014), while PIU emphasizes that it is a continuous variable with only degree differences (Lei and Yang, 2007). It shows that IA by poor parent-child relationship may be more symptomatic and not reach the level of disease, which also provides hope for intervention.

The IGDS is mainly used to measure IGA, which belongs to a special type of problematic Internet use and is developed according to the diagnostic criteria of IGA in DSM-5 (Pontes and Griffiths, 2015). Internet game addicts are more likely to be affected by negative emotions in cognitive expression, showing more depression, anxiety and hostility. Some researchers also believe that IGA is a “process addiction” mode, which refers to a person's severe dependence on a specific behavior (Lyu, 2017). This suggested that the association between IGA and the quality of parent-child relationship is stronger than that between the general internet addiction and the quality of parent-child relationship. When children indulge in Internet games, parents usually strengthen their regulation of the Internet to prevent problematic Internet use. However, a poor parent-child relationship led to weak supervision of online games by parents (Su et al., 2016). This indicated that when parent-child relationship quality is poor, children are more likely to enter the virtual game world and spend more time in it.

### Limitations and future research

The current study is the first systematic review of the association between the parent-child relationship and problematic Internet use since 1990. It can provide some guidance for future research, but there are some limitations.

#### Limitations

Several limitations of the current study should be noted.

First, all the studies included in the current study were cross-sectional studies, which makes it difficult to explore the causal association between the parent-child relationship and problematic Internet use. Longitudinal studies can be used in the future to explore the causal relationship between the two. Second, all the statistics are self-reported, which means there may be recall bias. In the future, we can add some studies rating by observers to compare whether there are differences. Third, although the random model was used in this study to minimize the impact of publication bias, attention should be paid to balancing the sample size in the future. Fourth, the number of countries included in this study was limited, so the findings may not apply to those not included. More studies in countries where studies on problematic Internet use are limited may enrich future studies. Fifth, the current meta-analysis only included studies related to children, adolescents and emerging adults, so the conclusions may not be applicable to older samples. Sixth, although this study divided problematic Internet use into five categories according to standards, there may also be conceptual overlap among different types of problematic Internet use. For example, Internet games may have functional overlap with social media (Laconi et al., 2017). In addition, WeChat, Facebook and other social media may also have game functions (Montag et al., 2015). Future studies need to clarify further the differences between different types of problematic Internet use, and include more types of problematic Internet use (e.g., problematic online shopping and pornography use) for research. Seventh, only one person in this study coded according to the coding standards at different times, so there may be misunderstandings. Eighth, this study did not strictly distinguish the gender difference between SMA and IGD, which may be the reason why the gender difference was insignificant in this study, and the two may be offset (Hawi and Samaha, 2019). A meta-analysis also found gender differences when the two were studied separately (Su et al., 2020). Finally, for three or more levels of moderating variables, the pair comparison method was used for the posttest, which may have increased the probability of α error and could be improved in future research.

#### Implications for practice and future study

a) Integrating studies that examined parent-child relationships from different aspects, it seems that compared with parent-child attachment and general types of parent-child relationships, parent-child communication was more important for the impact of problematic Internet use. This conclusion provides a direction for future intervention in the parent-child relationship and can further prevent the problematic Internet use by reducing the pressure on the surrounding socialenvironment.
b) Among different types of problematic Internet use, the association between SMA/IGD and the parent-child relationship was stronger, which indicated that it is necessary to further clarify the types of problematic Internet use and identify the differences among different types of problematic Internet use. A meta-analysis also found this difference (Su et al., 2020). Moreover, when the symptoms of problematic Internet use are not serious, appropriate intervention in the parent-child relationship will have a stronger protective effect. Future research could distinguish among the special types of problematic Internet use and the development stages of problematic Internet use.
c) This study did not strictly distinguish between father-child relationship and mother-child relationship between SMA and IGD. There were more females with SMA and more males with IGD (Su et al., 2020). A large number of studies have shown that same-sex parents have a greater impact on adolescents' problem behavior (Zhang et al., 2021). In the future, the differences between father and mother with different specific types of problematic Internet use can be further studied.
d) Studies on the association between emerging adults' problematic Internet use and the parent-child relationship should be strengthened. Emerging adults are in a stage of delayed identity, and when exploring life and self, the quality of social support (parent-child relationship) is very important to them. The availability of the Internet to emerging adults might also make them more prone to problematic Internet use. At the same time, it is also essential to strengthen the monitoring of problematic Internet use rates in countries with high power distance. The parent-child relationship may be more important, providing direction for futureintervention.
e) Currently, there are various scales for measuring parent-child relationship and problematic Internet use (Su and Lin, 2014), and their quality differs. When choosing scales, we should consider the types of problematic Internet use to be measured and the characteristics of parent-child relationship (Li et al., 2009), then choose the appropriate scale. Developing scales for different groups and different types to improve the quality of scale compilation.
f) The Internet use disorder model (Ceyhan et al., 2018) holds that problematic Internet use is bidirectionally related to personal factors (cognitive, emotional, and executive function), and the parent-child relationship may have an impact on personal factors and thus further affect Internet use disorder. Future research should explore the influence mechanism of parent-child relationship on individual factors and problematic Internet use can be furtherexplored.

## Conclusion

In conclusion, this study is the first to systematically examine the effect sizes of the association between parent-child relationship and problematic Internet use. Parent-child relationship was negatively correlated with problematic Internet use, and it was moderated by types of problematic internet use, age, country and scales of parent-child relationship and problematic internet use.

The current meta-analysis involving 114,098 subjects and 79 independent samples worldwide provides an accurate effect size of the differences from a global perspective. The association between social media addiction and parent-child relationship is stronger than the association between excessive Internet use and parent-child relationship. Moreover, the association between the parent-child relationship and problematic Internet use in emerging adults was significantly stronger than that in adolescents. Findings also suggested the association between the parent-child relationship and problematic Internet use was weaker in Italy than in Turkey or China. In addition, different measures have different effects on the association between the parent-child relationship and problematic Internet use. These results suggested that it is necessary to combine culture to define and study problematic Internet use in the future more accurately.

## Data availability statement

The original contributions presented in the study are included in the article/Supplementary material, further inquiries can be directed to the corresponding author/s.

## Author contributions

YZ: methodology, investigation, writing-original draft, data curation, formal analysis, visualization, writing-original draft, and writing-review & editing. LD: conceptualization, methodology, formal analysis, writing-original draft, writing-review & editing, project administration, and funding acquisition. KW: writing-original draft and writing-review & editing. All authors approval of the version to be published and agreement to be accountable for all aspects of the work.

## Conflict of interest

The authors declare that the research was conducted in the absence of any commercial or financial relationships that could be construed as a potential conflict of interest.

## Publisher's note

All claims expressed in this article are solely those of the authors and do not necessarily represent those of their affiliated organizations, or those of the publisher, the editors and the reviewers. Any product that may be evaluated in this article, or claim that may be made by its manufacturer, is not guaranteed or endorsed by the publisher.

## Footnotes

^1^ https://www.meta-analysis.com/
